# A systems medicine strategy to predict the efficacy of drugs for monogenic epilepsies

**DOI:** 10.1111/epi.17429

**Published:** 2022-10-25

**Authors:** Basel Taweel, Anthony G. Marson, Nasir Mirza

**Affiliations:** ^1^ Department of Pharmacology and Therapeutics, Institute of Systems, Molecular, and Integrative Biology University of Liverpool Liverpool UK

**Keywords:** Dravet syndrome, drug repurposing, epilepsy, genetics, genomics

## Abstract

**Objective:**

Monogenic epilepsies are rare but often severe. Because of their rarity, they are neglected by traditional drug developers. Hence, many lack effective treatments. Treatments for a disease can be discovered more quickly and economically by computationally predicting drugs that can be repurposed for it. We aimed to create a computational method to predict the efficacy of drugs for monogenic epilepsies, and to use the method to predict drugs for Dravet syndrome, as (1) it is the archetypal monogenic catastrophic epilepsy; (2) few antiseizure medications are efficacious in Dravet syndrome; and (3) predicting the effect of drugs on Dravet syndrome is challenging, because Dravet syndrome is typically caused by an *SCN1A* mutation, but some antiseizure medications that are efficacious in Dravet syndrome do not affect *SCN1A*, and some antiseizure medications that affect *SCN1A* aggravate seizures in Dravet syndrome.

**Methods:**

We have devised a computational method to predict drugs that could be repurposed for a monogenic epilepsy, based on a combined measure of drugs' effects upon (1) the function of the disease's causal gene and other genes predicted to influence its phenotype, (2) the transcriptomic dysregulation induced by the casual gene mutation, and (3) clinical phenotypes.

**Results:**

Our method correctly predicts drugs that are more effective, less effective, ineffective, and aggravating for seizures in people with Dravet syndrome. Our method correctly predicts the positive "hits" from large‐scale screening of compounds in an animal model of Dravet syndrome. We predict the relative efficacy of 1462 drugs. At least 38 drugs are ranked higher than one or more of the antiseizure drugs currently used for Dravet syndrome and have existing evidence of antiseizure efficacy in animal models.

**Significance:**

Our predictions are a novel resource for identifying new treatments for seizures in Dravet syndrome, and our method can be adapted for other monogenic epilepsies.

**Key Points**

- We devised a computational method to predict the efficacy of drugs for monogenic epilepsies such as Dravet syndrome
- Our method predicts drugs that are more effective, less effective, ineffective, and aggravating for Dravet syndrome
- Our predictions are a novel resource for identifying new treatments for seizures in Dravet syndrome
- Thirty‐eight of our predictions have published evidence of antiseizure efficacy in animal models
- Our method can be adapted to other monogenic epilepsies

## 1 INTRODUCTION

Monogenic epileptic encephalopathies are rare diseases characterized by early onset frequent drug‐resistant seizures that lead to devastating lifelong neurodisabilities.^1^ They are neglected diseases; their rarity makes traditional drug development methods uneconomical. Drug repurposing—finding treatments for a disease from among existing compounds already being used to treat other diseases—makes drug discovery for rare diseases viable. Drug repurposing for a disease can be accelerated by computationally predicting the drugs that are most likely to be efficacious for the disease.

Computational methods developed to predict drugs for polygenic diseases are unsuitable for monogenic diseases in general^2^, ^3^, ^4^, ^5^, ^6^ and monogenic epilepsies in particular. One strategy used to predict drugs for a polygenic disease is to identify the drugs that affect the function of genes underlying the disease. A monogenic disease is caused by a mutation in a single gene. However, knowing the identity of the mutant gene underlying a monogenic disease is not sufficient for predicting drugs for the disease. For example, Dravet syndrome, the archetypal monogenic epileptic encephalopathy, is typically caused by mutations in *SCN1A*. Some antiseizure medications that alleviate seizures in Dravet syndrome do not affect *SCN1A*, and some antiseizure medications that affect *SCN1A* aggravate seizures in Dravet syndrome.^7^ Another widely used method for predicting drugs is to identify compounds that could potentially reverse disease‐associated transcriptomic changes in disease‐affected tissue. Brain tissue from people with monogenic epilepsies is scarce/unavailable. Even when it is available, the transcriptomic profile has been altered by seizures and antiseizure medicines. Even the disease‐assisted transcriptomic profiles in animal model studies are typically contaminated with changes created by seizures. Finally, molecular/genetic/genomic drug prediction methods utilize data about drugs' effects that are derived from in vitro studies. A drug that exerts a desirable effect in vitro, might not be able to penetrate the blood–brain barrier and produce a clinical antiseizure effect in vivo.

There is a need for dedicated enhanced computational methods to predict drugs for monogenic diseases, in general and monogenic epilepsies in particular. We chose to focus on Dravet syndrome, because of the clinical need for more drugs to treat Dravet syndrome, and because of the utility of Dravet syndrome as a model to test the accuracy of our drug predictions. Dravet syndrome is a severe developmental epileptic encephalopathy. Few licensed or experimental antiseizure drugs are efficacious in people with or animal models of Dravet syndrome. Many of the currently licensed antiseizure drugs aggravate seizures in Dravet syndrome. Complete seizure control remains unattainable for most people with the condition, and the goal of current therapy is to reduce the frequency of seizures while minimizing the adverse effects of drugs. As such, it is desirable to identify additional drugs that can be used to treat Dravet syndrome, so that any individual with the condition has more treatment options available and, hence, better chances of finding an efficacious and well‐tolerated treatment. As the contrasting effects of different antiseizure drugs on Dravet syndrome are well studied and well recognized, the accuracy of drug predictions can be tested by determining whether they are consistent with clinical observations and studies. For the treatment of seizures in Dravet syndrome, different drugs can be classified as more effective, less effective, ineffective, and aggravating.^7^ Correctly categorizing drugs into these groups is a robust test of a drug prediction system.

## 2 MATERIALS AND METHODS

Technical methodological details can be found in the Supplementary Material.

## 3 RESULTS

### 3.1 Overview

To predict the relative efficacy of drugs for a monogenic disease, we created three distinct scores (Table 1), and then combined them to form our final Triple score.

**TABLE 1:** Methods created and used to predict drugs for Dravet syndrome

Drugs' effect on		Method name	Underlying hypothesis
#1	Causal genes	Genic Causality score	A drug is more likely to affect a disease if it affects the function of a gene that causes a more similar disease
#2	Dysregulated transcriptome	Transcriptomic Reversion score	A drug that is better at reversing disease‐associated transcriptomic changes is better at alleviating the disease
#3	Clinical phenotype	Clinical Effect score	A drug that is more likely to produce the adverse effects that are associated with antiseizure drugs is more likely to have antiseizure efficacy

*Note*: The methods are based upon drugs' effects on genic, transcriptomic, and clinical domains, as shown.

We evaluated the scores' ability to predict the following sets of drugs:

- Drugs that are more effective for Dravet syndrome;
- Drugs that are less effective for Dravet syndrome;
- Drugs that are ineffective for Dravet syndrome; and
- Drugs that are aggravating for Dravet syndrome.

The above drug sets were compiled from published literature (see Methods S1).

The scores' ability to predict these drug sets was evaluated using the following metrics:

- Identification: We used area under receiver operated characteristics curve (AUROC) analysis to determine how well our predictions identified drugs of interest (see Methods S1).
- Prioritization: How high are the drugs in a set ranked on average? This is the median rank of the drugs in the set, expressed as a percentile. For example, if a drug set has a prioritization score of 90%, this means that the median‐ranked drug in the set is ranked higher than 90% of all drugs.
- Enrichment: How much are all the drugs in a set enriched at the top of the drug predictions? For example, if all of the drugs in a drug set are ranked within the top 10% of predictions, this equates to a 10× enrichment. The statistical significance of the enrichment is calculated using the hypergeometric equation.

### 3.2 Genic Causality score

This score is based upon the following premise: A drug is more likely to affect a phenotype if it affects the function of a gene that causes a more similar phenotype.

Every gene was ascribed a causality score, based on the similarity to Dravet syndrome of the phenotype produced by a mutation in the gene (Table 2). The Genic Causality score of each drug was the sum of the causality scores of all the genes it changes in function.

**TABLE 2:** All genes (whose protein products are expressed in the brain and changed in function by drugs) were categorized and scored as shown

#	Gene category	Details	Enrichment	Score
I	Causes Dravet syndrome	*SCN1A*	53	53
II	Cause Dravet‐like syndrome	Genes that cause clinical phenotypes identical/similar to Dravet syndrome	26	26
III	Cause epileptic encephalopathy	Genes that cause epileptic encephalopathies	19	19
IV	Cause monogenic epilepsy	Genes that cause monogenic epilepsies	14	14
V	Enriched in brain	Genes with ≥4× higher mRNA level in brain compared to any other tissue	8	8
VI	Elevated in brain	Genes with ≥4× higher average level of mRNA expression in a group of 2–5 tissues, including brain, compared to all other tissues or ≥4× higher mRNA level in brain compared to the average level in all other tissues	3	3
VII	Expressed in brain	Genes expressed in the human brain	1	1

*Note*: Enrichment is the fold enrichment of the antiseizure drugs that affect Dravet syndrome among all the drugs that affect the function of any gene in each category. Enrichment is calculated relative to all the drugs that affect the function of any gene across the human genome. Except for Category VII, all enrichments were statistically significant (false discovery rate‐corrected hypergeometric equation *p* < .05). Monogenic epilepsy and epileptic encephalopathy genes (phenotypic series PS308350 and PS617711) were collated from Online Mendelian Inheritance in Man (www.omim.org). Genes expressed, elevated, and enriched in brain, as identified and defined by the Human Protein Atlas,^21^ were collated from their website (www.proteinatlas.org/humanproteome/brain/human+brain). All weblinks were accessed on November 13, 2020.

By random permutation of the causality scores ascribed to each gene, we showed that the metrics achieved by our scoring system are unlikely to be observed by chance (permutation‐based *p* < 1 × 10^−3^).

Drugs with a higher Genic Causality score have a stronger effect on Dravet syndrome. The Genic Causality score prioritizes and enriches the antiseizure drugs that are more effective for Dravet syndrome (Table 3). However, as the Genic Causality score lacks directionality—it cannot predict whether a drug exerts an alleviating or aggravating effect—it also prioritizes and enriches the antiseizure drugs that aggravate Dravet syndrome. The antiseizure drugs that are less effective for Dravet syndrome are prioritized and enriched less than both the more effective and the aggravating antiseizure drugs. Hence, the Genic Causality score is good at distinguishing the antiseizure drugs that are more effective for Dravet syndrome from the antiseizure drugs that are less effective for Dravet syndrome, but not as good at distinguishing the antiseizure drugs that are more effective for Dravet syndrome from the antiseizure drugs that are aggravating for Dravet syndrome.

**TABLE 3:** Results obtained using scores individually, in pairwise combinations and in triple combination

Metric	Drugs	Triple	GC + TR	GC + CE	TR + CE	GC	TR	CE
Enrichment	More effective	55.1	45.4	33.5	9.9	34.3	3.6	8.1
	Less effective	1	1	3	1	2.3	1.4	2.6
	Ineffective	1	1	1	1	1	1	1
	Aggravating	1	1	4.4	1	2.5	1.1	16.8
Prioritization	More effective	99.5	99.4	99.1	98.4	98.6	88.2	96
	Less effective	97	97	97.1	85.5	96.6	65.8	90.1
	Ineffective	52.9	52.1	44.7	52.8	50.3	51.9	34.4
	Aggravating	.6	1.8	98.7	1.8	96.8	25.7	97.1
Identification	More effective	—	—	—	—	—	—	—
	Less effective	.864	.862	.763	.868	.782	.901	.719
	Ineffective	1	.998	1	.981	.998	.83	.998
	Aggravating	.888	.89	.615	.946	.72	1	.337

*Note*: Enrichment is fold change. For example, more effective drugs are enriched at the top of the Triple score predictions 55.1× more than expected by chance. Prioritization is the median rank of the drugs in the set, expressed as a percentile. For example, if a drug set has a prioritization score of 90%, this means that the median‐ranked drug in the set is ranked higher than 90% of all drugs. Identification is the mean AUROC value for distinguishing "more effective" drugs from the other drugs shown. More detailed results, including hypergeometric equation *p*‐values for enrichment and SDs for AUROC, can be found in Table S1.

Abbreviations: AUROC, area under receiver operated characteristics curve; CE, Clinical Effect score; GC, Genomic Causality score; TR, Transcriptomic Reversion score.

### 3.3 Transcriptomic Reversion score

This method is based on the premise that drugs that are better at reversing disease‐associated transcriptomic changes are better at alleviating the disease.^6^ Briefly, disease‐associated^8^ and drug‐induced transcriptomic changes^9^ were compared, to predict each drug's relative ability to reverse disease‐associated transcriptomic changes. Drugs that induce transcriptomic changes more strongly inverse of the disease‐associated transcriptomic changes are predicted to be more effective against the disease. The transcriptome‐based drug efficacy predictions were performed using the Combination Connectivity Mapping bioconductor package and the Library of Integrated Network‐Based Cellular Signatures (LINCS) data,^9^ as previously described.^10^ This package utilizes cosine distance as the (dis)similarity metric. A higher (more negative) cosine distance value indicates that the drug induces gene‐expression changes more strongly opposed to those associated with the disease. A lower (more positive) cosine distance value indicates that the drug induces gene‐expression changes more similar to those associated with the disease.

Drugs with a favorable Transcriptomic Reversion score have an ameliorating effect on Dravet syndrome, whereas drugs with an unfavorable Transcriptomic Reversion score have an aggravating effect on Dravet syndrome. Drug predictions based on the transcriptome of Dravet syndrome are concordant with the clinically observed effects of drugs on Dravet syndrome; the antiseizure drugs that are more effective for Dravet syndrome are predicted to be more effective, the antiseizure drugs that are less effective for Dravet syndrome are predicted to be less effective, and the antiseizure drugs that are aggravating for Dravet syndrome are predicted to be aggravating (Table 3). In contrast, drug predictions based on transcriptomes not of Dravet syndrome are not concordant with the clinically observed effects of drugs on Dravet syndrome (Table 4).

**TABLE 4:** Results for "more effective" drugs if measured TR scores for "more effective" drugs are used in the Triple score versus if imputed TR scores for "more effective" drugs are used in the Triple score

Scores	Identification			Prioritization	Enrichment
	Less effective	Ineffective	Aggravating		
With imputed TR values	.86 ± .10	1.0 ± 0	.89 ± .1	99.5	59.3×
With measured TR values	.86 ± .11	1.0 ± 0	.89 ± .1	99.5	55.1×

*Note*: Enrichment is fold change. For example, more effective drugs are enriched at the top of the measured predictions 55.1× more than expected by chance. Prioritization is the median rank of the drugs in the set, expressed as a percentile. For example, if a drug set has a prioritization score of 90%, this means that the median‐ranked drug in the set is ranked higher than 90% of all drugs. Identification is the area under receiver operated characteristics curve (mean ± SD) value for distinguishing "more effective" drugs from the other drugs shown.

Abbreviation: TR, Transcriptomic Reversion.

This method prioritizes and enriches the antiseizure drugs that are more effective for Dravet syndrome more, and the antiseizure drugs that are less effective for Dravet syndrome less (Table 3). Importantly, as this method possesses directionality—it can predict if a drug exerts an alleviating or aggravating effect—it also deprioritizes the antiseizure drugs that aggravate Dravet syndrome and depletes, from the top predictions, the antiseizure drugs that aggravate Dravet syndrome. This method perfectly distinguishes the antiseizure drugs that are more effective for Dravet syndrome from the antiseizure drugs that aggravate Dravet syndrome.

### 3.4 Clinical Effect score

The Clinical Effect score is premised upon the following postulates. Certain adverse clinical effects are more likely to be produced by antiseizure drugs than by drugs with no antiseizure efficacy and, hence, can be used to distinguish antiseizure drugs from drugs with no antiseizure efficacy. Drugs that are more likely to produce the adverse effects that better distinguish antiseizure drugs from drugs with no antiseizure efficacy are more likely to have antiseizure efficacy. Briefly, we downloaded data for every adverse event report for every drug in the US Food and Drug Administration's Adverse Event Reporting System for postmarketing safety surveillance (https://www.fda.gov/drugs/questions‐and‐answers‐fdas‐adverse‐event‐reporting‐system‐faers/fda‐adverse‐event‐reporting‐system‐faers‐public‐dashboard; accessed June 1, 2021), creating a novel dataset of >35 million individual adverse event reports and the associated drugs (available upon request). This dataset was used to calculate (1) the relative ability of each adverse effect to distinguish antiseizure drugs from drugs with no antiseizure efficacy and (2) the relative likelihood that each drug produces each adverse effect. These data were then integrated to predict the relative likelihood of antiseizure efficacy for each drug as follows. If:

1. The adverse effects produced by *Drug D* are labeled *1* … *n*; and
2. *D*_*n*_ is the relative likelihood that *Drug D* produces adverse effect *n*; and
3. *n*_*asm*_ is a relative measure of how well adverse effect *n* distinguishes antiseizure drugs from drugs with no antiseizure efficacy,

then, the Clinical Effect score for *Drug D* can be represented by:

$$\sum_{n}^{1}\left(D_{n}\times n_{\mathrm{asm}}\right)$$

The Clinical Effect score distinguishes drugs with antiseizure efficacy from drugs with no antiseizure efficacy. The Clinical Effect score identifies, prioritizes, and enriches the antiseizure drugs that are effective for Dravet syndrome (Table 3). This method perfectly distinguishes the antiseizure drugs that are more effective for Dravet syndrome from the antiseizure drugs that have no antiseizure efficacy. The antiseizure drugs more effective for Dravet syndrome receive favorable Clinical Effect scores even if they are excluded from the analysis performed to measure adverse effects' ability to distinguish antiseizure drugs from drugs with no antiseizure efficacy; identification, prioritization, and enrichment values were 1.0 ± .02, 96.3, and 7.8×, respectively, when they were excluded, and 1.0 ± .01, 96.0, and 8.1×, respectively, when they were included.

### 3.5 Triple score

The three scores above were combined to create a Triple score. Briefly, the absolute values of the three scores were rescaled between 1 and 100, and then multiplied.

**FIGURE 1:**
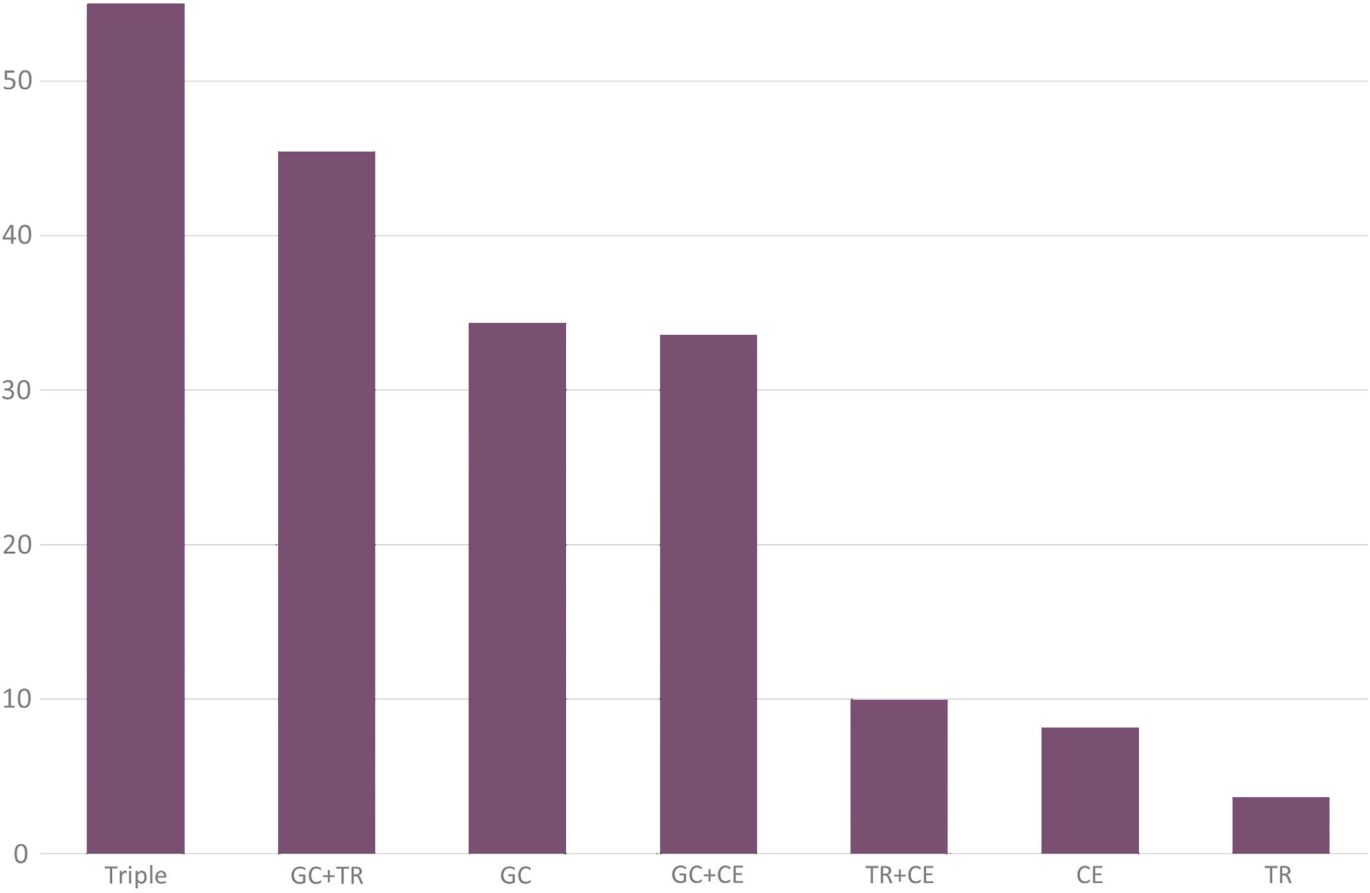
Enrichment of antiseizure drugs that are more effective for Dravet syndrome among the predictions from different individual scores and combinations of scores. CE, Clinical Effect score; GC, Genic Causality score; TR, Transcriptomic Reversion score

The antiseizure drugs more effective for Dravet syndrome are prioritized and enriched more by the Triple score method than by any individual or pairwise combination of methods (Table 3 and Figure 1). The Triple score prioritizes the antiseizure drugs more effective for Dravet syndrome > the antiseizure drugs less effective for Dravet syndrome > the drugs ineffective for Dravet syndrome > the antiseizure drugs that aggravate Dravet syndrome. The Triple score distinguishes the antiseizure drugs that are more effective for Dravet syndrome from the antiseizure drugs that are less effective, ineffective, or aggravating for Dravet syndrome. The Triple score discriminates perfectly the antiseizure drugs more effective for Dravet syndrome from the drugs ineffective for Dravet syndrome.

### 3.6 Imputing missing Transcriptomic Reversion scores to generate Triple scores for more drugs

Computation of the Transcriptomic Reversion score requires profiles of drug‐induced transcriptomic changes assayed in the LINCS program and provided in their database.^9^ The Transcriptomic Reversion score and, hence, the Triple score cannot be calculated for a drug that has not been assayed in the LINCS program. Both Genic Causality scores and Clinical Effect scores can be calculated for 1462 drugs; Transcriptomic Reversion scores cannot be calculated for 691 (47%) of these drugs, as they have not been assayed in the LINCS program. To generate Triple scores for the drugs that have not been assayed in the LINCS program, we imputed their Intermediate Transcriptomic Phenotype scores from their Genic Causality scores and their Clinical Effect scores.

To estimate the accuracy of the imputation, we deleted the measured Transcriptomic Reversion scores for the more effective drugs, and then imputed them. The identification, prioritization, and enrichment of the more effective drugs achieved using the imputed scores was at least as good as that achieved using measured scores (Table 4).

An alternative strategy to generate combined scores for more drugs is to discard the Transcriptomic Reversion score altogether. We found that imputing the missing Transcriptomic Reversion scores gives better results than discarding all the Transcriptomic Reversion scores (Table S2).

The measured or imputed scores for all drugs are provided in Table S3.

### 3.7 Potential for accelerating the discovery of effective drugs from in vivo screening

The largest screening of compounds in an animal model of Dravet syndrome has been performed by Baraban et al. (https://barabanlab.ucsf.edu/drug‐discovery‐database; accessed March 1, 2022). Using the zebrafish model of Dravet syndrome, they have screened a large number of compounds, 1337 of which overlap with the drugs in our databases. If our in silico method is used to rank these compounds by their predicted order of efficacy for Dravet syndrome, after excluding any compounds with missing Intermediate Transcriptomic Phenotype scores, 100% of the positive hits from the in vivo screening are found in the top ~7% of the computationally ranked compounds, which is a ~14‐fold and statistically significant enrichment (hypergeometric *p* = 1.5 × 10^−6^). If our in silico method is used to rank these compounds by their predicted order of efficacy against Dravet syndrome, after imputing any missing Intermediate Transcriptomic Phenotype scores, 100% of the positive hits from the in vivo screening are found in the top ~14% of the computationally ranked compounds, which is a ~7‐fold and statistically significant enrichment (hypergeometric *p* = 1.1 × 10^−6^).

### 3.8 Examples of promising predicted drugs

We focus on drugs that are ranked higher than the lowest ranked first‐ or second‐line antiseizure drugs for Dravet syndrome. Our predictions include drugs that are licensed for conditions other than epilepsy but have published evidence of antiseizure efficacy in animal models of epilepsy. At least 38 such drugs are ranked higher than the lowest ranked first‐ or second‐line antiseizure drugs for Dravet syndrome. Twenty‐three of these drugs have evidence from multiple published studies and/or animal models. These drugs and their supporting published studies are shown in Table S4, alongside their current clinical indication.

## 4 DISCUSSION

We have developed a novel computational method to predict drugs' relative efficacy against a monogenic epilepsy. We have used the method to predict drugs for Dravet syndrome. Our method significantly prioritizes and enriches drugs that are effective for Dravet syndrome in clinical trials and experience and in experimental studies. Our predictions correctly rank the sets of drugs that are more effective, less effective, ineffective, and aggravating for Dravet syndrome.

Our predictions are a valuable resource for selecting candidate drugs that could be used for treating seizures in Dravet syndrome. Our predictions include relatively new antiseizure drugs—brivaracetam, felbamate, perampanel, and zonisamide—that have not been tested in clinical trials for Dravet syndrome and for whom there is limited experience in Dravet syndrome. Case studies, case series, and clinical experience indicate that these drugs are potentially efficacious for Dravet syndrome.^11^, ^12^, ^13^, ^14^ Hence, these drugs merit study in clinical trials for Dravet syndrome. Our predictions also include drugs that are licensed for conditions other than epilepsy but have published evidence of antiseizure efficacy in animal models of epilepsy. At least 38 such drugs are ranked higher than the lowest ranked first‐ or second‐line antiseizure drugs for Dravet syndrome. These drugs and their supporting published studies are shown in Table S4, alongside their current clinical indication. These data can be used to select for further study candidate drugs that could potentially have an antiseizure effect and address other important health needs of people with Dravet syndrome. For example, >70% of people with Dravet syndrome have sleep problems.^15^ One of the drugs predicted to have antiseizure efficacy in Dravet syndrome is glutethimide, which was developed to treat insomnia. Glutethimide has an antiseizure effect in two different animal models.^16^ People with Dravet syndrome can display agitation and aggression.^17^ Antipsychotics aripiprazole and haloperidol might be useful for treating these symptoms and seizures, as both are predicted to have antiseizure efficacy in Dravet syndrome, and have evidence of antiseizure efficacy from four different studies in four different animal models. Candidate drugs will have to be validated in future human clinical trials before being deployed in clinical practice.

Our aim was to create a system that optimizes the prediction of drugs for monogenic epilepsies by utilizing a wide range of publicly available data. We created methods, applicable to monogenic epilepsies, based on drugs' effects upon the function of causal proteins, the abundance of intermediate transcripts, and the expression of clinical symptoms. By definition, a monogenic epilepsy is caused by a single gene mutation. However, we devised a novel method of weighting all brain‐expressed genes according to their likelihood of affecting a monogenic phenotype, and using these data to predict drugs. Utilizing a disease's associated transcriptome to predict drugs for the disease is an established technique. Importantly, we show that *SCN1A* mutation‐induced genome‐wide transcriptomic dysregulation must be assayed in brain tissue from Dravet syndrome model animals that have become susceptible to but have not yet developed seizures, as seizure‐induced transcriptomic changes will likely confound *SCN1A* mutation‐induced transcriptomic changes, once seizures have developed. This observation is likely to hold true for other monogenic epilepsies also. The Clinical Effect score is a novel method for predicting drugs that are likely to have a clinical antiseizure effect. The three scores utilize orthogonal data and provide results with complementary strengths. Combining all three methods leads to better performance than any individual or pairwise combination of these methods. Alongside these strengths, our method has some limitations, discussed below.

The Genic Causality score relies upon knowledge of the proteins changed in function by drugs. At present, knowledge of the proteins that are changed in function by each drug is incomplete, and it is more incomplete for some drugs than for others. The more incomplete the knowledge of the proteins changed in function by a drug, the more likely it is that the drug's Genic Causality score will be underestimated. By extension, the Genic Causality score is more likely to be underestimated for drugs that are less studied, as their modes of action are less analyzed and, hence, knowledge of the proteins changed in function by them is less complete. Nevertheless, drug predictions can only be based upon available data, and the drugs with the higher Genic Causality scores are the ones that affect the higher number of causal genes, according to available data. Furthermore, the drug prediction performance achieved by the Genic Causality score cannot be achieved if the genes are ascribed random weights, which suggests that the Genic Causality score's drug prediction performance cannot be explained solely by the sheer number of genes affected by each drug.

*SCN1A* mutation‐induced genome‐wide transcriptomic dysregulation was assayed in tissue from an animal model rather than from people with Dravet syndrome. It is not known how closely the transcriptomic changes in this animal model of Dravet syndrome recapitulate the transcriptomic changes in people with Dravet syndrome. However, this animal model reproduces the clinical features and response to antiseizure drugs seen in people with Dravet syndrome, which suggests that its transcriptomic profile reproduces the transcriptomic profile of people with Dravet syndrome. For some drugs, the intermediate transcriptome score is not currently available, as their transcriptomic effects are still to be assayed. By imputing missing intermediate transcriptome score values, the number of drugs whose efficacy is predicted can be expanded. Whereas the magnitude of the intermediate transcriptome score can be imputed, its directionality cannot. As a result, drug prediction performance of the imputed dataset is lower. Despite this, imputing the missing intermediate transcriptome score values produces better drug prediction results than omitting the intermediate transcriptome score entirely from the drug prediction framework.

The Clinical Effect score is a novel method that distinguishes drugs with clinical antiseizure efficacy from drugs with no clinical antiseizure efficacy. The Clinical Effect score is not specific to Dravet syndrome, but it achieves perfect discrimination between drugs that are efficacious against seizures in people with Dravet syndrome and drugs with no antiseizure efficacy in people. Adverse effect profile similarity between drugs has been used to predict drugs that could be repurposed for new indications, based on the precept that drugs with similar adverse effect profiles are likely to be efficacious for the same disease.^18^, ^19^, ^20^ Adverse effect profiles are constructed from the adverse effects listed in published literature, which typically do not provide data for the likelihood of an adverse effect being produced by a drug. Hence, the adverse effects that are very likely to be produced by a drug and the adverse effects that are very unlikely to be produced by the drug are weighted equally in its adverse effect profile. Our clinical phenotype score is the first method for performing drug repurposing analysis by exploiting the vast amount of data found in a national adverse effects reporting database. We use these data to estimate the relative likelihood of every adverse effect being produced by each drug. Our drug predictions are based on the relative likelihood that a drug produces each of its reported adverse effects, and how well each adverse effect differentiates antiseizure drugs from drugs with no clinical antiseizure efficacy. The Clinical Effect score might be biased against less commonly used and newer drugs, as there is less chance for their adverse effects to be reported and less time for the reports to accumulate. Nevertheless, drug predictions can only be based upon available data.

Whereas the Transcriptomic Reversion score predicts directionality of drugs' effect (alleviating or aggravating), the Genic Causality and Clinical Effect scores do not. The combined Triple score differentiates alleviating from aggravating drugs better than the Genic Causality and Clinical Effect scores, but not as well as the Transcriptomic Reversion score, which achieves perfect discrimination between alleviating and aggravating antiseizure drugs for Dravet syndrome. When calculating the final combined Triple score, its three constituent weights were weighted equally. In future analyses, the weightings can be altered, giving greater weight to particular constituent score(s), to optimize the drug prediction performance according to the preferences/needs of the researchers/project. For example, greater weighting of the Transcriptomic Reversion score will decrease the yield of potentially aggravating drugs, while increasing the yield of drugs that might not penetrate the brain and exert a clinical effect.

There is increasing interest in medium throughput animal model drug screening using lower animal species. Our findings suggest that using our method to create a shortlist of candidate drugs for medium throughput animal model screening can potentially increase the yield of the animal model screening manyfold, decreasing the time and resource cost of the animal model screening. Furthermore, efficacy of a drug in lower animal species does not indicate that the drug is human blood–brain barrier permeable, but a drug preselected using our computational method is more likely to penetrate the brain and produce a clinical antiseizure effect in people.

Our method is potentially generalizable to other epileptic encephalopathies, other monogenic epilepsies, and other monogenic diseases. Its applicability to another disease will depend on the availability of genetic information about the disease and an appropriate animal model of the disease. If these prerequisites are fulfilled, this method could potentially be used to select candidate drug(s) for animal model screening and, if successful, clinical trials.

## AUTHOR CONTRIBUTIONS

Nasir Mirza conceived the study. Nasir Mirza and Basel Taweel designed the study. Nasir Mirza and Basel Taweel performed data analyses. All authors interpreted the data. All authors drafted and approved the final version of the manuscript.

## CONFLICT OF INTEREST

The authors declare that they have no competing interests. We confirm that we have read the Journal's position on issues involved in ethical publication and affirm that this report is consistent with those guidelines.

## Supporting information


Tables S1–S4

Methods S1
