# Supplementary material for: A systems medicine strategy to predict the efficacy of drugs for monogenic epilepsies
Source: Epilepsia. 2022 Oct 25;63(12):3125–33. doi: 10.1111/epi.17429 (PMC10092251; doi:10.1111/epi.17429)
Supplement: Supplementary file 2 — Methods S1 [file EPI-63-3125-s002.docx]

**Supplementary methods**

**The Genic Causality Score**

We hypothesised that: A drug is *more* *likely* to affect Dravet syndrome if it affects the function of genes that cause diseases that are *more like* Dravet syndrome. Our hypothesis is based on the following reasoning:

- A drug is likely to affect a disease if it affects another similar disease; the more similar the disease, the higher the likelihood.
- A drug is likely to affect a disease if it affects the gene(s) underlying the disease.
- Therefore, a drug is likely to affect a disease if it affects the gene(s) underlying another similar disease; the more similar the disease, the higher the likelihood.

Genes were divided into seven categories, ranging from the most likely (*SCN1A*) to the least likely to cause diseases like Dravet syndrome (Table 2).

Genes in category II (cause Dravet-like syndrome) were identified through a comprehensive search and review of published literature. The results of our literature review were concordant with a subsequently published systematic review of published literature for the identification of genes that cause “Dravet syndrome and Dravet syndrome-like phenotypes”.^1^ Some patients diagnosed with Dravet syndrome *clinically* have subsequently been found to carry a mutation in one of these genes. Hence, mutations in these genes can produce phenotypes that are *almost* identical to Dravet syndrome, but often subtly different, for example, as discussed by Larsen and colleagues for *SCN8A*.^2^

Three of the categories contain genes that are not known to cause monogenic epilepsies/encephalopathies: genes expressed, elevated and enriched in the human brain. The biological justification of their inclusion and order in our scoring system is as follows. Genes in these categories are not known to cause epilepsies, but are likely to contain yet undiscovered epilepsy-causing genes, with undiscovered epilepsy-causing genes being more enriched in brain-enriched genes than in brain-elevated genes than in brain-expressed genes. Compared to the set of genes expressed in the brain, the set of genes elevated in the brain is >2.2-fold enriched with genes that are known to cause monogenic epilepsies (hypergeometric distribution p-value < 6.0e-11). Compared to the set of genes expressed in the brain, the set of genes enriched in the brain is >5.6-fold enriched with genes that are known to cause monogenic epilepsies (hypergeometric distribution p-value < 9.2e-15). This suggests that brain-enriched genes, more than brain-elevated genes, more than brain-expressed genes are likely to be causally-relevant to epilepsy.

The drugs that affect the function of genes in each successive category (from least to most likely to cause epilepsies like Dravet syndrome) are progressively more enriched with antiseizure drugs that affect Dravet syndrome (Table 2). Every gene was ascribed a score equal to the fold enrichment of antiseizure drugs that affect Dravet syndrome amongst all the drugs that affect the function of any gene in its category. As alternative scoring systems, score increments ranging from 10% to 100% were applied to each successive gene category, from least to most likely to cause epilepsies like Dravet syndrome. These alternative scoring systems yielded similar results and the same conclusion (data not shown).

The Genic Causality score for each drug is the sum of the scores of all the genes it changes in function.

**The Transcriptomic Reversion score**

This method is based on the premise that drugs that are better at reversing disease-associated transcriptomic changes are better at treating the disease. In this method, drug-induced transcriptomic changes are compared with disease-associated transcriptomic changes. Drugs that induce transcriptomic changes more strongly inverse of the disease-associated transcriptomic changes are predicted to be more effective against the disease. As seizures cause widespread transcriptomic changes, we postulated that the Dravet syndrome brain tissue used for the transcriptomic analysis should not be affected by recent seizures. The transcriptome was obtained from a published RNA-Seq analysis of hippocampi from a mouse model of Dravet syndrome.^3^ Specifically, we extracted genes differentially expressed between the hippocampi of wildtype and *Scn1a*^+/−^ mice of an epilepsy-susceptible strain, after the age of seizure-onset, but **without** recent seizures. To validate the use of this model for predicting the efficacy of drugs in Dravet syndrome, we compared the drug predictions obtained by using its transcriptome and by using other models’ transcriptomes. The comparator transcriptomes were obtained as follows:

- We extracted, from the same study, the transcriptomes of three other types of *Scn1a*^+/−^ mice:
  - *Scn1a^+/−^* mice of an epilepsy-susceptible strain, after age of seizure-onset, **with** recent seizures. This is the transcriptome of Dravet syndrome, contaminated with transcriptomic changes induced by seizures.
  - *Scn1a^+/−^* mice of an epilepsy-susceptible strain, before age of seizure-onset. This is the transcriptome of mutant mice that are susceptible to but have not yet developed Dravet syndrome.
  - *Scn1a^+/−^* mice of an epilepsy-resistant strain. This is the transcriptome of mutant mice that are resistant to Dravet syndrome.
- Gene-expression changes associated with human focal epilepsy were imputed from the results of the most recent genome-side association study (GWAS) for epilepsy,^4^ as previously described.^5^ (We opted not to use the generalised epilepsy GWAS results as some of the antiseizure drugs that aggravate seizures in Dravet syndrome can also aggravate some types of generalised seizures.)

The mouse model transcriptomes used in our analysis comprised all genes identified as significantly differentially expressed (FDR <0.05) in the published results. The only exception was the transcriptome of mutant mice that are resistant to Dravet syndrome, as only a single gene is differentially expressed in this strain; for this strain, we used gene expression signatures of the same length as for *Scn1a^+/−^* mice of the epilepsy-susceptible strain, after the age of seizure-onset, but without recent seizures. The mouse genes were mapped to human homologues listed in the Mouse Genome Database.^6^

The transcriptome-based drug efficacy predictions were performed using the Combination Connectivity Mapping bioconductor package and the Library of Integrated Network-Based Cellular Signatures (LINCS) data,^7^ as previously described.^4^ This package utilizes cosine distance as the (dis)similarity metric. A higher (more negative) cosine distance value indicates that the drug induces gene-expression changes more strongly opposed to those associated with the disease. A lower (more positive) cosine distance value indicates that the drug induces gene-expression changes more similar to those associated with the disease.

**The Clinical Effect score**

The Clinical Effect score is premised upon the following:

1. There are certain adverse effects that are more likely to be produced by antiseizure drugs than by drugs with no antiseizure efficacy.
2. These adverse effects can be used to identify other drugs with antiseizure efficacy: drugs that are more likely to produce the adverse effects that distinguish antiseizure drugs from drugs with no antiseizure efficacy are more likely to have antiseizure efficacy.

If:

1. the adverse effects produced by *Drug D* are labelled *1 . . . n*, and
2. *D_n_* is the relative likelihood that *Drug D* produces adverse effect *n*, and
3. *n_asm_* is a relative measure of how well adverse effect *n* distinguishes antiseizure drugs from drugs with no antiseizure efficacy,

then, the CP score for *Drug D* can be represented by:

$$\sum_{n}^{1} \left( D_{n}\times n_{asm} \right)$$

*D_n_* is the relative likelihood that *Drug D* produces adverse effect *n*. The calculation of *D_n_* is based on the following precept: *Drug D* is more likely than *Drug C* to produce adverse effect *n* if reports of adverse effect *n* are more enriched amongst the total reports of *Drug D's* adverse effects, than they are amongst the total reports of *Drug C's* adverse effects. The enrichment of each adverse effect amongst the total reports of each drug’s adverse effects was calculated as follows.

All FDA Adverse Event Reporting System (FAERS) Quarterly Data Extract Files (<https://www.fda.gov/drugs/questions-and-answers-fdas-adverse-event-reporting-system-faers/fda-adverse-event-reporting-system-faers-latest-quarterly-data-files>) were downloaded, up to and including the fourth quarter of 2020. Preparations with drug combinations were excluded. Where multiple drugs were listed in the same case report, only the ‘primary suspect’ drug was retained. In FAERS, drug names do not conform to a standardized nomenclature: some drugs are listed using their generic name, some using a brand name, and some with the formulation, dose or manufacturer’s name, etc, appended to the drug name. All brand names were converted to their generic names shown in the FDA Orange Book (<https://www.fda.gov/media/76860/download>; accessed 01/06/2021). Differing forms of generic drug names were standardized to the names shown in the WHO ATC dataset (<https://www.genome.jp/kegg-bin/get_htext#A1>; accessed 01/06/2021). Our final processed adverse events dataset includes >35 million individual adverse event reports, and the associated drugs (available upon request). This dataset was used to calculate the enrichment of each adverse effect amongst the total reports of each drug’s adverse effects. Statistical significance of enrichment, rather than *fold* enrichment, was used, as any drug with very few total adverse effect reports can have some adverse effects with seemingly very high fold enrichments, which might not be a true reflection of the relative likelihood of the drug producing that adverse effect. Enrichment p-values were calculated using the hypergeometric equation. Enrichment p-values and the raw numbers used to calculate them for all unique drug-adverse effect pairs are available upon request.

*n_asm_* is a relative measure of how well adverse effect *n* distinguishes antiseizure drugs from drugs with no antiseizure efficacy. First, for each drug in the database, the relative likelihood that it produces adverse effect *n* is calculated as described above. Then, these values are used to calculate the AUROC for adverse effect *n*’s ability to distinguish antiseizure drugs from drugs with no antiseizure efficacy. The AUROC for adverse effect *n* is the relative measure of how well it distinguishes antiseizure drugs from drugs with no antiseizure efficacy (*n_asm_*). The adverse effects that are more likely to be caused by the antiseizure drugs more effective for Dravet syndrome might get better *n_asm_* scores because antiseizure drugs more effective for Dravet syndrome are amongst the drugs used to calculate *n_asm_* scores. This might bias the Clinical Phenotype Score in favour of the antiseizure drugs that are more effective for Dravet syndrome. To determine if this is the case, Clinical Phenotype Scores were recalculated after if excluded antiseizure drugs more effective for Dravet syndrome from the drugs used to calculate *n_asm_* scores.

**The ‘Triple Score’**

The three scores above were combined to create a Triple Score. In order to give each of the three constituent scores equal weighting in the combined Triple Score, the absolute values of the three scores were rescaled between 1 and 100. After this, the triple score was calculated as indicated below:

The Triple Score for *Drug D* = *r*CG*_D_* × *r*IT*_D_* × *r*CP*_D_*

Where *r*CG*_D_*, *r*IT*_D_* and *r*CP*_D_* are the rescaled Causal Gene score, the rescaled Transcriptomic Reversion score and the rescaled Clinical Phenotype score for *Drug D*, respectively.

**Imputing missing Transcriptomic Reversion scores to generate Triple Scores for more drugs**

In order to generate Triple Scores for the drugs that have not been assayed in the LINCS programme, we imputed their Transcriptomic Reversion scores from their Causal Gene scores and their Clinical Phenotype scores. *k*-nearest neighbour imputation was performed using *simputation* (version 0.2.7; <https://cran.r-project.org/web/packages/simputation/index.html>). The value of *k* that optimises the identification, prioritisation and enrichment of more effective antiseizure drugs was identified by comparing the results obtained with values of *k* between 1 and 20. Imputation was repeated 1000×, to generate 1000 Triple Score values; the mean of these values was taken as the final imputed Triple Score. The magnitude of the Transcriptomic Reversion is imputable, but not its directionality (beneficial or deleterious). All imputed values were assumed to be in a positive/beneficial direction, accepting that this will not be the case for all drugs. An alternative strategy to generate ‘Triple Scores’ for more drugs is to discard the Transcriptomic Reversion score altogether; we also calculated the results obtained using this approach.

**Categorisation of drugs:**

Drugs were divided into the following categories:

- More effective drugs: First- and second-line antiseizure drugs for Dravet syndrome, according to published expert consensus statements or expert multiauthor reviews. The published expert consensus statements or expert multiauthor reviews were identified by performing a Medline search on the 25^th^ of September, 2020, for multiauthor review articles that had Dravet AND (treatment OR management) in the title.^8-15^
- Less effective drugs: all antiseizure drugs that are neither more effective nor aggravating for Dravet syndrome. We have previously compiled the list of all antiseizure drugs.^5^
- Ineffective drugs: Drugs that do not cross the human blood-brain barrier and, hence, are ineffective for Dravet syndrome. This list of drugs was compiled from published studies.^16-29^
- Aggravating drugs: antiseizure drugs that can aggravate seizures in Dravet syndrome, according to expert consensus statements or expert multiauthor reviews. The published expert consensus statements or expert multiauthor reviews were identified as described above.

**Area under receiver operated characteristics curve analysis**

We used area under receiver operated characteristics curve (AUROC) analysis to determine the accuracy with which drugs’ scores discriminate antiseizure drugs from all other drugs, or discriminate more from less clinically-effective subsets of antiseizure drugs. AUROC was calculated using the package PRROC (version 1.3.1)^30^ in R (version 3.4.3). In assessing the discrimination of ‘more effective’ antiseizure drugs from other drugs, there is a class imbalance, because a small number of drugs are ‘more effective’ antiseizure drugs. To correct for this imbalance, we employed the standard technique of random under-sampling.^5^ Briefly, AUROC was calculated using the set of ‘more effective’ antiseizure drugs and a randomly selected set from the other drugs equal in number to the ‘more effective’ antiseizure drugs. This process was repeated 1000 times, and mean (± standard deviation) AUROC was calculated.

**References for supplementary methods**

- 1 Ding, J. *et al.* Do All Roads Lead to Rome? Genes Causing Dravet Syndrome and Dravet Syndrome-Like Phenotypes. *Front Neurol* **13**, 832380, doi:10.3389/fneur.2022.832380 (2022).
- 2 Larsen, J. *et al.* The phenotypic spectrum of SCN8A encephalopathy. *Neurology* **84**, 480-489, doi:10.1212/WNL.0000000000001211 (2015).
- 3 Hawkins, N. A., Calhoun, J. D., Huffman, A. M. & Kearney, J. A. Gene expression profiling in a mouse model of Dravet syndrome. *Exp Neurol* **311**, 247-256, doi:10.1016/j.expneurol.2018.10.010 (2019).
- 4 International League Against Epilepsy Consortium on Complex, E. Genome-wide mega-analysis identifies 16 loci and highlights diverse biological mechanisms in the common epilepsies. *Nat Commun* **9**, 5269, doi:10.1038/s41467-018-07524-z (2018).
- 5 Mirza, N. *et al.* Using common genetic variants to find drugs for common epilepsies. *Brain Commun* **3**, fcab287, doi:10.1093/braincomms/fcab287 (2021).
- 6 Eppig, J. T. *et al.* The Mouse Genome Database (MGD): facilitating mouse as a model for human biology and disease. *Nucleic Acids Res* **43**, D726-736, doi:10.1093/nar/gku967 (2015).
- 7 Subramanian, A. *et al.* A Next Generation Connectivity Map: L1000 Platform and the First 1,000,000 Profiles. *Cell* **171**, 1437-1452 e1417, doi:10.1016/j.cell.2017.10.049 (2017).
- 8 Wheless, J. W., Fulton, S. P. & Mudigoudar, B. D. Dravet Syndrome: A Review of Current Management. *Pediatr Neurol* **107**, 28-40, doi:10.1016/j.pediatrneurol.2020.01.005 (2020).
- 9 Cross, J. H. *et al.* Dravet syndrome: Treatment options and management of prolonged seizures. *Epilepsia* **60 Suppl 3**, S39-S48, doi:10.1111/epi.16334 (2019).
- 10 Wirrell, E. C. & Nabbout, R. Recent Advances in the Drug Treatment of Dravet Syndrome. *CNS Drugs* **33**, 867-881, doi:10.1007/s40263-019-00666-8 (2019).
- 11 Ziobro, J., Eschbach, K., Sullivan, J. E. & Knupp, K. G. Current Treatment Strategies and Future Treatment Options for Dravet Syndrome. *Curr Treat Options Neurol* **20**, 52, doi:10.1007/s11940-018-0537-y (2018).
- 12 Knupp, K. G. & Wirrell, E. C. Treatment Strategies for Dravet Syndrome. *CNS Drugs* **32**, 335-350, doi:10.1007/s40263-018-0511-y (2018).
- 13 Wirrell, E. C. *et al.* Optimizing the Diagnosis and Management of Dravet Syndrome: Recommendations From a North American Consensus Panel. *Pediatr Neurol* **68**, 18-34 e13, doi:10.1016/j.pediatrneurol.2017.01.025 (2017).
- 14 Wallace, A., Wirrell, E. & Kenney-Jung, D. L. Pharmacotherapy for Dravet Syndrome. *Paediatr Drugs* **18**, 197-208, doi:10.1007/s40272-016-0171-7 (2016).
- 15 Chiron, C. & Dulac, O. The pharmacologic treatment of Dravet syndrome. *Epilepsia* **52 Suppl 2**, 72-75, doi:10.1111/j.1528-1167.2011.03007.x (2011).
- 16 Toropov, A. A., Toropova, A. P., Beeg, M., Gobbi, M. & Salmona, M. QSAR model for blood-brain barrier permeation. *J Pharmacol Toxicol Methods* **88**, 7-18, doi:10.1016/j.vascn.2017.04.014 (2017).
- 17 Gao, Z., Chen, Y., Cai, X. & Xu, R. Predict drug permeability to blood-brain-barrier from clinical phenotypes: drug side effects and drug indications. *Bioinformatics* **33**, 901-908, doi:10.1093/bioinformatics/btw713 (2017).
- 18 Castillo-Garit, J. A., Casanola-Martin, G. M., Le-Thi-Thu, H., Pham-The, H. & Barigye, S. J. A Simple Method to Predict Blood-Brain Barrier Permeability of Drug- Like Compounds Using Classification Trees. *Med Chem* **13**, 664-669, doi:10.2174/1573406413666170209124302 (2017).
- 19 Fan, J., Yang, J. & Jiang, Z. Prediction of Central Nervous System Side Effects Through Drug Permeability to Blood-Brain Barrier and Recommendation Algorithm. *J Comput Biol* **25**, 435-443, doi:10.1089/cmb.2017.0149 (2018).
- 20 Wang, Z. *et al.* In Silico Prediction of Blood-Brain Barrier Permeability of Compounds by Machine Learning and Resampling Methods. *ChemMedChem* **13**, 2189-2201, doi:10.1002/cmdc.201800533 (2018).
- 21 Yuan, Y., Zheng, F. & Zhan, C. G. Improved Prediction of Blood-Brain Barrier Permeability Through Machine Learning with Combined Use of Molecular Property-Based Descriptors and Fingerprints. *AAPS J* **20**, 54, doi:10.1208/s12248-018-0215-8 (2018).
- 22 Gupta, M., Lee, H. J., Barden, C. J. & Weaver, D. F. The Blood-Brain Barrier (BBB) Score. *J Med Chem* **62**, 9824-9836, doi:10.1021/acs.jmedchem.9b01220 (2019).
- 23 Miao, R., Xia, L. Y., Chen, H. H., Huang, H. H. & Liang, Y. Improved Classification of Blood-Brain-Barrier Drugs Using Deep Learning. *Sci Rep* **9**, 8802, doi:10.1038/s41598-019-44773-4 (2019).
- 24 Saxena, D., Sharma, A., Siddiqui, M. H. & Kumar, R. Blood Brain Barrier Permeability Prediction Using Machine Learning Techniques: An Update. *Curr Pharm Biotechnol* **20**, 1163-1171, doi:10.2174/1389201020666190821145346 (2019).
- 25 Wang, Y. *et al.* An experimentally validated approach to calculate the blood-brain barrier permeability of small molecules. *Sci Rep* **9**, 6117, doi:10.1038/s41598-019-42272-0 (2019).
- 26 Loscher, W. & Friedman, A. Structural, Molecular, and Functional Alterations of the Blood-Brain Barrier during Epileptogenesis and Epilepsy: A Cause, Consequence, or Both? *Int J Mol Sci* **21**, doi:10.3390/ijms21020591 (2020).
- 27 Singh, M., Divakaran, R., Konda, L. S. K. & Kristam, R. A classification model for blood brain barrier penetration. *J Mol Graph Model* **96**, 107516, doi:10.1016/j.jmgm.2019.107516 (2020).
- 28 SABER, R., RIHANA, S. & MHANNA, R. in *2019 Fifth International Conference on Advances in Biomedical Engineering (ICABME).* 1-4 (IEEE).
- 29 Alsenan, S., Al-Turaiki, I. & Hafez, A. A Recurrent Neural Network model to predict blood-brain barrier permeability. *Comput Biol Chem* **89**, 107377, doi:10.1016/j.compbiolchem.2020.107377 (2020).
- 30 Grau, J., Grosse, I. & Keilwagen, J. PRROC: computing and visualizing precision-recall and receiver operating characteristic curves in R. *Bioinformatics* **31**, 2595-2597, doi:10.1093/bioinformatics/btv153 (2015).
